# Li_4_(OH)_3_Br-Based Shape Stabilized Composites for High-Temperature TES Applications: Selection of the Most Convenient Supporting Material

**DOI:** 10.3390/nano11051279

**Published:** 2021-05-13

**Authors:** Imane Mahroug, Stefania Doppiu, Jean-Luc Dauvergne, Angel Serrano, Elena Palomo del Barrio

**Affiliations:** 1 Centre for Cooperative Research on Alternative Energies (CIC energiGUNE), Basque Research and Technology Alliance (BRTA), Alava Technology Park, 01510 Vitoria-Gasteiz, Spain; sdoppiu@cicenergigune.com (S.D.); jldauvergne@cicenergigune.com (J.-L.D.); aserrano@cicenergigune.com (A.S.); epalomo@cicenergigune.com (E.P.d.B.); 2 INP Bordeaux, UMR 5295, I2M, CNRS, Avenue Pey-Berland 16, 33607 Pessac, France; 3 UMR 5295, I2M, CNRS, University of Bordeaux, Esplanade des Arts et Métiers, 33405 Talence, France; 4 Department of Applied physics, University of the Basque Country UPV-EHU, 48940 Leioa, Spain; 5 Ikerbasque, Basque Foundation for Science, 48013 Bilbao, Spain

**Keywords:** peritectic compound Li_4_(OH)_3_Br, phase change materials, thermal energy storage, shape stabilized composites, supporting materials, oxides

## Abstract

Peritectic compound Li_4_(OH)_3_Br has been recently proposed as phase change material (PCM) for thermal energy storage (TES) applications at approx. 300 °C Compared to competitor PCM materials (e.g., sodium nitrate), the main assets of this compound are high volumetric latent heat storage capacity (>140 kWh/m^3^) and very low volume changes (<3%) during peritectic reaction and melting. The objective of the present work was to find proper supporting materials able to shape stabilize Li_4_(OH)_3_Br during the formation of the melt and after its complete melting, avoiding any leakage and thus obtaining a composite apparently always in the solid state during the charge and discharge of the TES material. Micro-nanoparticles of MgO, Fe_2_O_3_, CuO, SiO_2_ and Al_2_O_3_ have been considered as candidate supporting materials combined with the cold-compression route for shape-stabilized composites preparation. The work carried out allowed for the identification of the most promising composite based on MgO nanoparticles through a deep experimental analysis and characterization, including chemical compatibility tests, anti-leakage performance evaluation, structural and thermodynamic properties analysis and preliminary cycling stability study.

## 1. Introduction

High-temperature thermal energy storage (HT-TES) is part of the storage solution that is expected to be deployed in future energy systems. HT-TES is currently used in concentrating solar thermal power plants to warrant dispatchability. Moreover, using HT-TES is also envisaged in conventional thermal power plants to provide them with greater operational flexibility. It is also expected for HT-TES to provide a second life to coal-fired plants, which are being closed for environmental reasons, and that participate as well in the emergence of stand-alone energy storage plants in the grid, where it has a cost advantage over other technologies. In the industrial sector, in addition to the already known uses of recovery and valorization of waste heat and improvement of the overall efficiency of cogeneration systems or steam boilers, the use of HT-TES associated with increasing solarization and/or electrification of heat and cold production will be added.

The development of HT-TES technologies has been closely linked to the development and deployment of concentrating solar thermal power plants [1,2]. The technology that currently dominates the market is molten salt due to the ability of nitrate mixtures to operate at temperatures up to 450–560 °C at reasonable cost, despite the drawbacks of the risk of salt solidification and inherent corrosion problems. At lower temperatures (up to 400 °C), thermal storage in concrete is also a commercially available option. Fixed packed-bed systems, which use rocks or solid industrial by-products as granular filler material, are in the process of being commercialized. Compared to molten salts and concrete, their main advantages are much lower investment costs and the possibility to operate in a very wide temperature range (up to 1000 °C).

All of the above technologies store/deliver energy by increasing/decreasing the temperature of the storage material (sensible heat storage) and therefore suffer from a lack of compactness (low storage capacity per unit volume). In this regard, latent heat storage technologies based on high-temperature phase change materials (HT-PCMs) could meet the desired compactness objectives while keeping costs affordable. Inorganic anhydrous salts and their mixtures are the most commonly investigated HT-PCMs [3,4,5,6]. They store thermal energy in an almost isothermal manner during melting, and they return it back during the reverse process of solidification. Moreover, they are usually characterized by high enthalpies of fusion, high density, excellent thermochemical stability and low price. However, they often suffer from some shortcomings. The most important ones are corrosivity and low thermal conductivity (<1 W/m/K). Corrosion involves using expensive corrosion-resistant materials for the storage tank and heat exchanger, while low thermal conductivity has to be compensated by oversizing the latter, thus increasing investment cost.

To overcome such problems, different techniques for encapsulating high-temperature salts are being investigated. They can be classified into two main categories, namely core–shell microencapsulation [7,8,9,10,11], where the shell acts as a container to prevent liquid leakage, and so-called shape-stabilized composite materials (ss-composites) [8,9,11,12,13], where a porous supporting material encapsulates the salt and retain the liquid phase by capillary forces and surface tension. Compared with core–shell microencapsulation, ss-composites have clear advantages regarding production cost and performance. Indeed, they are generally produced by simple melting infiltration in a porous support or by cold compression of a mixture of the supporting material micro and/or nanoparticles and salt powders. Moreover, they usually have the ability of self-management of the volume changes of the salt during phase transitions, which is one of the major concerns of core–shell microencapsulation, and they lead to higher apparent thermal conductivity enhancement.

The selection of the supporting material is critical for successful ss-composites [12]. The thermal stability in the planned working temperature range and the chemical compatibility with encapsulated salt are the most basic requirements, which directly determine the usability of the supporting material. Good wettability with loaded salt and high specific surface area is also of great importance because it determines maximum salt loading and, therefore, the latent heat storage capacity of the final material. To a lesser extent, high thermal conductivity is also advisable to reduce the size of heat exchangers or, alternatively, to maximize the size of pellets/grains in fixed packed-bed storage systems. Obviously, economic and safety aspects are also relevant, therefore, safe (non-corrosive, non-flammable, non-explosive) and inexpensive supporting materials that are easy to obtain and process are required as well.

Supporting material used in ss-composites studied so far can be classified into three main groups:

- Carbon-based supporting materials, such as expanded graphite and graphite foams [14,15,16,17,18,19,20,21,22,23,24,25,26,27,28]. They have proven to be compatible with nitrites and chlorides and have high salt absorption capacity (>85 wt.%). Furthermore, they are excellent in heat transfer enhancement because of their high thermal conductivity (up to 100 W/m/K reported). However, they are characterized by poor wettability with salts, and they also tend to oxidize at temperatures above approx. 600 °C. The ss-composites using expanded graphite are usually prepared by the uniaxial or isostatic cold-compression route, whereas the vacuum-assisted melting infiltration method is used in the preparation of graphite foam-based composites.
- Clay mineral supporting materials such as expanded perlite, expanded vermiculite and diatomite [9,29,30,31,32,33,34,35,36]. They demonstrate chemical compatibility with nitrites, chlorides and sulfates as well as high salt absorption capacity (>85 wt.% for expanded perlite and vermiculite; 55–70 wt.% for diatomite). Moreover, the wettability with molten salts is good, and they can support temperatures above 1000 °C. However, they have low values of thermal conductivity (<0.15 W/m/K), and the melting infiltration route is needed for ss-composite preparation, which is more expensive than the cold compression method. Another type of clay mineral used as an additive for PCM composites is natural halloysite nanoclay. These materials are characterized by good thermal stability, a high adsorption capacity and low cost. Halloysite nanoclay is used generally as a nucleating agent to mitigate the supercooling phenomena of the hydrate PCMs and is applied for cold storage [37,38]
- Other supporting materials including refractory oxides (MgO, Al_2_O_3_, SiO_2_, mullite), SiC and Ca(OH)_2_ [11,13,39,40,41,42,43,44,45,46,47,48,49,50,51,52,53,54,55,56,57]. The compatibility and good wettability with nitrates, carbonates, chlorides and sulfates have been proven for most of them. Maximum salt loading is lower than in previous cases, but still significant (up to 70 wt.%). On the contrary, they show excellent thermal stability up to 1400–1600 °C (only 570 °C for calcium hydroxide). In addition, they have relatively high thermal conductivity (3–65 W/m/K), and corresponding ss-composites are prepared by the cold-compression route.

The present work deals with the peritectic compound Li_4_(OH)_3_Br recently proposed as an HT-PCM for TES applications at approx. 300 °C [58,59,60]. Compared to sodium nitrate, which is the reference HT-PCM for this temperature level [61], Li_4_(OH)_3_Br has two main advantages that make it particularly attractive [60]. On the one hand, the volumetric latent heat storage capacity of Li_4_(OH)_3_Br (141.3 kWh/m^3^) is 54% higher than that of NaNO_3_, meaning that Li_4_(OH)_3_Br offers the opportunity for reducing significantly the volume of the storage tank. On the other hand, whereas the volume expansion on melting of NaNO_3_ is quite high (almost 11%), that of Li_4_(OH)_3_Br is only 3%. This work aims at selecting suitable supporting materials for Li_4_(OH)_3_Br-based ss-composites obtained by the cold-compression route, which is the most advantageous from an economic point of view. Candidate supporting materials considered are micro and/or nanoparticles of MgO, Fe_2_O_3_, CuO, SiO_2_ and Al_2_O_3_. An experimental screening, including chemical compatibility with Li_4_(OH)_3_Br analysis, anti-leakage performance and maximum salt loading evaluation as well as thermal cycling stability of corresponding ss-composites, was performed to select the best candidate.

## 2. Materials and Methods

### 2.1. Materials

High purity anhydrous lithium hydroxide (CAS: 1310-65-2, purity 98%) and lithium bromide (CAS: 7550-35-8, purity 99+%), both provided by Acros Organics (Geel, Belgium), were used in the preparation of the peritectic compound Li_4_(OH)_3_Br. The synthesis was performed following the method proposed by Mahroug et al. [60]. A powder mixture of LiOH and LiBr (5 g approx.) was prepared under a protective argon atmosphere by weighing the right weight fraction of each component (mole ratio 75LiOH:25LiBr) using a Sartorius balance (±0.1 mg). The mixture was then homogenized by ball milling for 15 min using a Spex mixer mill (875 rpm, Spexsampleprep, Metuchen, NJ, USA) using stainless-steel vials and stainless-steel balls (3 balls of 3 mm BPR = 0.5) under mild conditions. The mixture was then introduced inside corundum crucibles put inside stainless steel reactors and sealed under argon. The synthesis was performed inside the furnace applying the following temperature program: (i) a heating ramp at 10 °C/min from ambient temperature up to 30 °C above the melting temperature of Li_4_(OH)_3_Br; (ii) an isothermal step of 1 h; (iii) a cooling step up to room temperature with a cooling rate of 1.8 °C/min. Key thermophysical properties of Li_4_(OH)_3_Br are gathered in Table 1.

As supporting material for shape stabilization, several commercial oxides were tested with different particle sizes (nano/microparticles). General information about the tested materials is presented in Table 2.

**Figure 1:**
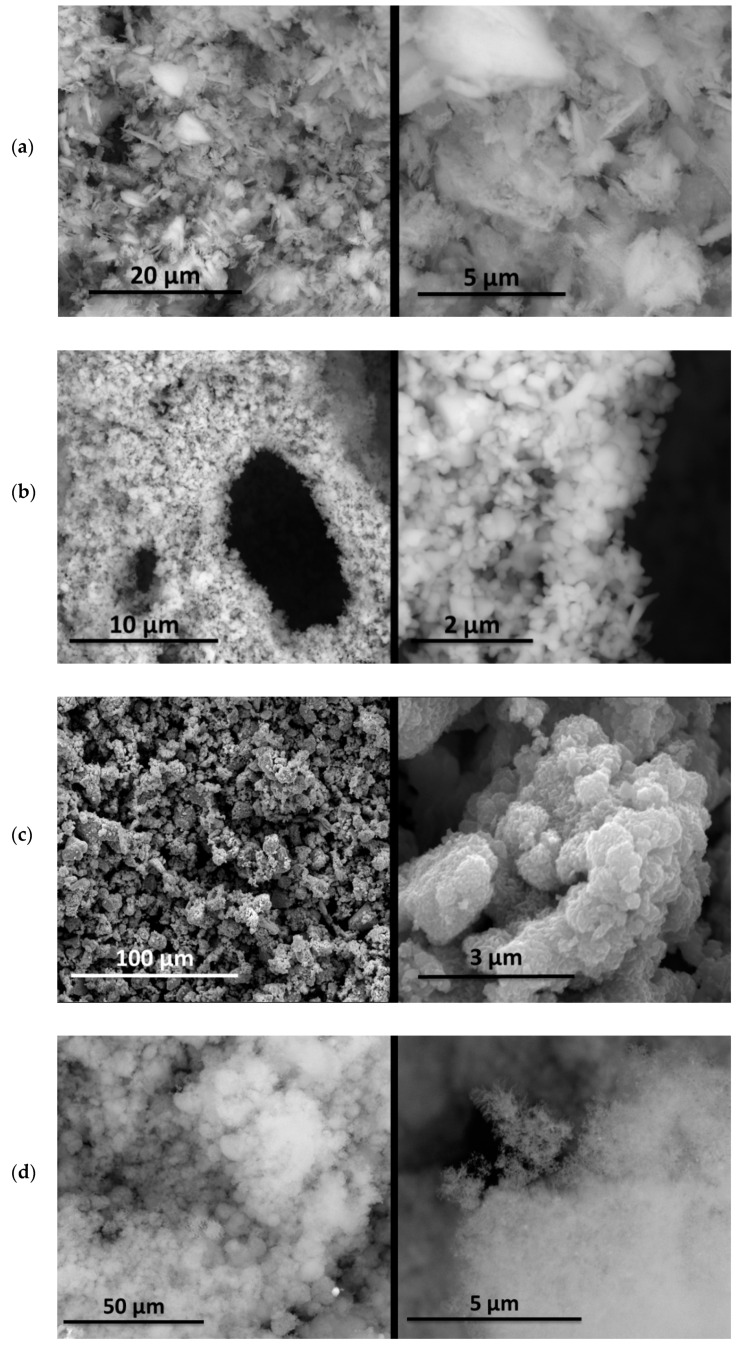

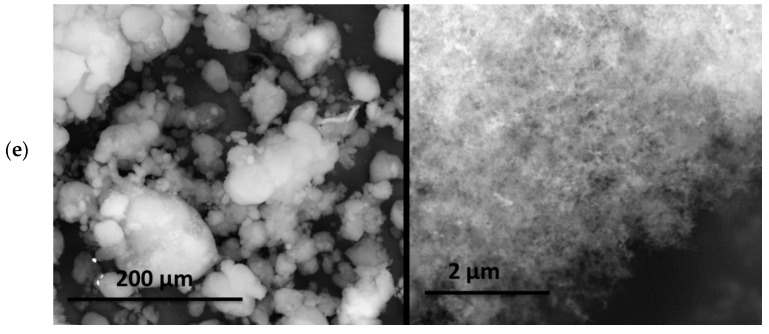
SEM images of the tested oxides: (**a**) MgO; (**b**) Fe_2_O_3_; (**c**) CuO; (**d**) Al_2_O_3_; (**e**) SiO_2_.

The morphology of the supporting materials was studied by Scanning Electron Microscopy (SEM) using a Quanta 200 FEG (FEI, Hillsboro, OR, USA) scanning electron microscope operated in high vacuum mode at 20 kV, with a back scattered electron detector (BSED). Particles size and morphology of the oxide supporting materials (MgO, Fe_2_O_3_, CuO, Al_2_O_3_ and SiO_2_) were studied by analyzing SEM images of the samples using ImageJ 2.0 software [62]. As can be seen in Figure 1, Al_2_O_3_ and SiO_2_ nanopowders formed spherical clusters with well-distributed cluster sizes (Figure 1d and Figure 1e, respectively); the particle size provided by the supplier was considered in the study. In the case of MgO nanopowder, Figure 1a shows crystal agglomerates of MgO with crystal size less than 1 µm. Fe_2_O_3_ (see Figure 1b) presented spherical powder with uniform distribution of the particle size (<4 µm), whereas CuO showed a large distribution of particle size (<13 µm).

Li_4_(OH)_3_Br-based composites were prepared by the uniaxial cold compression route. The storage material Li_4_(OH)_3_Br was initially grounded and sifted using a 200 µm sieve, and then it was mixed with the oxide according to specific mass ratios to obtain a total mass of 1 g of composite. The powder mixture was then physically mixed for 20 min using a ball mill (Spex mixer mill 875 RPM) without balls. The purpose of the physical mixing was to ensure the homogeneity of the oxide/salt mixture. Finally, a pellet of 13 mm diameter was made by cold compression of the powder mixture under a pressure of 5 tons for 1 min. The pellet was then placed in a corundum crucible inside a closed stainless steel reactor under Ar atmosphere and sintered in a muffle furnace over the melting temperature of the salt according to the following temperature program: a first heating step at 10 °C/min up to 350 °C, followed by an isothermal step at 350 °C for 1 h, and finally the sample was cooled down to room temperature at around 2 K/min cooling rate.

### 2.2. Screening Methodology

The experimental screening carried out to select the best supporting material focused on fundamental aspects such as chemical compatibility of the supporting material with Li_4_(OH)_3_Br, anti-leakage performance of the corresponding ss-composite and maximum salt loading allowed as well thermal properties and thermal cycling stability of the final composites. A three steps methodology was established to progressively discard either useless or poorly performing supporting material:

1. Chemical compatibility test. It consists of preparing a mixture of 90 wt.% Li_4_(OH)_3_Br and 10 wt.% oxide. The powder mixture is then subjected to a heating process up to 400 °C (Tm_salt_ + 60 °C) for 24 h inside a closed stainless-steel reactor under Ar atmosphere. After these extreme heating conditions, chemical compatibility is investigated by means of differential scanning calorimetry (DSC) analysis and X-ray diffraction analysis to detect eventual side-reactions or changes/degradations in the storage properties of Li_4_(OH)_3_Br.
2. Anti-leakage performance analysis and maximum salt loading allowed. Pellets of Li_4_(OH)_3_Br/oxide composite materials with different oxide loadings are prepared following the cold-compression method described in Section 2.2. They are then submitted to the following thermal treatment: a heating step at 10 °C/min up to 350 °C, followed by an isothermal step at 350 °C for 1 h and finally a cooling step at around 2 K/min up to room temperature. The effectiveness of the composite in retaining the liquid phase of Li_4_(OH)_3_Br is qualified by visual inspection of the pellets during the test. Those composites allowing higher salt content while displaying good anti-leakage performance are moved to the last step.
3. Stability of the composites under thermal cycling conditions. In this step, the phase transition properties of composites that passed previous tests are determined before and after 50 heating and cooling cycles. Thermal cycling tests are carried out in a muffle furnace under argon atmosphere, between 250 °C and 350 °C, applying heating/cooling rates of 10 K/min and 2 K/min, respectively. Determination of both cycling and storage properties are carried out using differential scanning calorimetry (TA DSC 2500 model (New Castle, DE, USA)). The composite showing better stability and heat storage capacity is finally selected as supporting material.

### 2.3. Thermal and Structural Characterizations

Thermal properties including reaction temperature and enthalpy were measured using differential scanning calorimetry (TA DSC 2500 model). The DSC measurements were performed for samples in the form of cohesive solids to preserve the shape stabilization effect. Samples of about 20 mg were analyzed at a heating rate of 1 °C/min and a cooling rate of 10 K/min in the temperature range of 40–300 °C. Hermetically sealed aluminum DSC crucibles were used for the measurement. The temperature and enthalpy were calibrated using sapphire and indium standards; argon was used as a purge gas (50 mL/min). The accuracy was estimated to be ±1 K for the temperature and ±3 J/g for the enthalpy. The phase transition temperature was considered as the onset temperature. The phase change enthalpy was calculated by peak integration in the heating run. Structural analysis of the materials was performed by X-ray diffraction analysis using a Bruker D8 Discover (Billerica, MA, USA) equipped with a LYNXEYE detector with monochromatic Cu Kα_1_ radiation of λ = 1.54056 Å. Patterns were recorded in a 2θ angular range 10–80° with a step size of 0.02° and a step time of 1 s. The measurements were performed at room temperature.

## 3. Results

**Figure 2:**
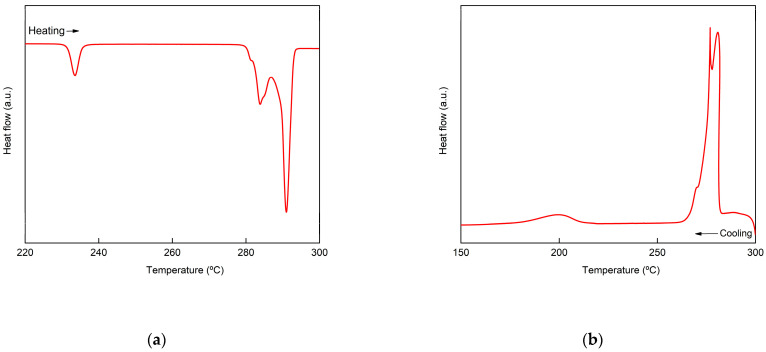
DSC curves of Li_4_(OH)_3_Br (**a**) upon heating and (**b**) upon cooling.

The peritectic salt Li_4_(OH)_3_Br was deeply studied in a previous paper as TES material [60]. The study showed that upon heating, Li_4_(OH)_3_Br undergoes different reversible phase transitions. A first solid state transformation occurs at around 230 °C. A second solid state transition occurs at 279 °C, and finally the peritectic reaction occurs at 289 °C. All these transformations are reflected in the DSC curve of this stoichiometric compound, as shown in Figure 2. This is the reference DSC in terms of transition temperature and reaction enthalpy, which were taken into consideration when analyzing the compatibility and performance of the different supporting materials. The values assigned to the peritectic reaction in this work represent the sum of both the second solid state transition and the peritectic transition.

### 3.1. Chemical Compatibility of the Supporting Material with Molten Li_4_(OH)_3_Br

**Figure 3:**
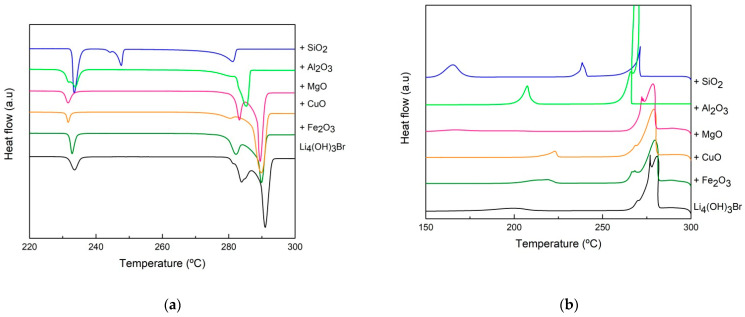
DSC curves of Li_4_(OH)_3_Br/oxide mixtures after compatibility tests recorded at 1 °C/min (**a**) upon heating and (**b**) upon cooling.

As already mentioned in Section 2.2 (step 1 of the screening), different mixtures of Li_4_(OH)_3_Br/oxide were prepared using 10 wt.% of the support materials reported in Table 2 and heated at 400 °C for 24 h inside closed stainless-steel reactors under Ar atmosphere. Then, all the samples were subjected to structural analysis using X-ray diffraction in order to test the compatibility with the salt and detect possible side products formed due to the reaction between the salt and the oxide. The DSC curves of different mixtures (Li_4_(OH)_3_Br + 10 wt.% oxide) after compatibility tests obtained both upon heating and cooling are reported in Figure 3.

The temperatures and enthalpies of the reaction corresponding to the different composites are presented in Table 3. The enthalpy of the peritectic reaction relative to the mass fraction of the salt in the composite Δ*H*_calculated_ is calculated as follows:

(1) $$\Delta H_{\mathrm{Calculated}}=\Delta H_{{\mathrm{Li}}_{4}{\left(\mathrm{OH}\right)}_{3}\mathrm{Br}}*X_{{\mathrm{Li}}_{4}{\left(\mathrm{OH}\right)}_{3}\mathrm{Br}}$$

where $X_{{\mathrm{Li}}_{4}{\left(\mathrm{OH}\right)}_{3}\mathrm{Br}}$ represents the mass fraction of the salt, and $\Delta H_{{\mathrm{Li}}_{4}{\left(\mathrm{OH}\right)}_{3}\mathrm{Br}}$ is the enthalpy of the pure peritectic salt.

Analyzing Figure 3a, the DSC curves of sample 90Li_4_(OH)_3_Br-10SiO_2_ showed a narrowing of the peak at the peritectic transition temperature together with the broadening of the first peak, in addition to the appearance of a new DSC peak at 246 °C. The three thermal events were reversible upon cooling (see Figure 3b). This behavior is an indication of a chemical reaction between the salt and the silica nanopowder. For this reason, the silicon dioxide was discarded at this level. Alumina nanopowder was also discarded due to the huge loss of the enthalpy of the peritectic reaction (−34%), as shown in Table 3. In addition, the DSC heating curve showed the displacement of both the second solid state reaction and the peritectic reaction to lower temperatures (−7 °C). The reason for this phenomenon may be due to the very small particle size of Al_2_O_3_ powder. In fact, the use of nanometric power (13 nm) with a very high specific surface area will create a large number of cavities inside the composite pellet, which leads to the possible confinement of a quantity of the peritectic salt inside these cavities, inducing the Gibbs–Thomson effect. Additionally, the salt trapped inside the cavities and the vacancies can cause a total or partial suppression of the peritectic reaction, which may be the reason for the significant decrease in the peritectic reaction enthalpy. In the cases of CuO, MgO and Fe_2_O_3_ oxides, good thermal stability of the composites was noticed. The temperature of the peritectic phase transition remained unchanged. Considering the systematic error (± 3 J/g), the enthalpy of the peritectic reaction was stable in the case of the Li_4_(OH)_3_Br/CuO composite; however, it showed a slight decrease (6% loss) in the cases of MgO and (10% loss) Fe_2_O_3_ composites. CuO, MgO and Fe_2_O_3_ metal oxides were selected for further investigation (shape stability performance and thermal cycling stability).

**Figure 4:**
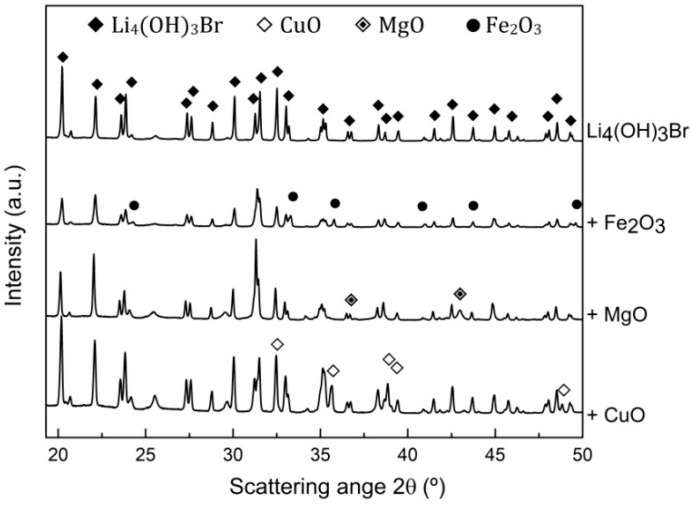
XRD results of Li_4_(OH)_3_Br/oxide mixtures after compatibility tests.

Analyzing the XRD results of Li_4_(OH)_3_Br and the composite materials reported in Figure 4, it can be clearly seen that the patterns corresponding to the samples 90Li_4_(OH)_3_Br-10Fe_2_O_3_, 90Li_4_(OH)_3_Br-10MgO, 90Li_4_(OH)_3_Br-10CuO showed only the peaks related to the peritectic salt plus the Fe_2_O_3_, MgO and CuO, respectively. No new peaks related to the formation of new phases were detected despite the harsh conditions applied for the compatibility tests, which indicates good chemical compatibility between the salt and the tested oxides.

### 3.2. Anti-Leakage Effectiveness and Maximum Salt Loading

Capillary forces are mainly responsible for retaining the liquid salt during the phase change process. The parameters that determines the capillary effect are the size and topology of the pores, as well as the adhesion forces between the liquid salt and the pore wall, the latter of which is estimated by the effective contact angle between the liquid salt and the supporting material (the higher the liquid adhesion, the lower the effective contact angle) [63,64]. The work required to displace a liquid tube outside a cylindrical pore is given by

(2) $$dW\equiv \Delta P_{c}\times dV=\frac{2\gamma_{lv}\cos\theta }{r}dV=S_{p}\gamma_{\mathrm{lv}}\cos\theta dV$$

where $\Delta P_{c}$ is the capillary pressure, dV is the infinitesimal volume of the liquid tube displaced along the axis of a cylindrical pore of radius *r*, $\gamma_{\mathrm{lv}}$ is the liquid–vapor surface tension, $S_{p}=2/r$ represents the surface area per unit of pore volume and *θ* is the effective contact angle. It can be concluded that the greater the wettability and the higher the surface area (smaller pore size for same total porosity), the better the anti-leakage efficiency.

From this, several conclusions of practical interest follow for the interpretation of the results of the leakage tests performed: (1) decreasing the size of the oxide particles at constant oxide loading improves anti-leakage efficiency of the ss-composite; (2) the same happens when increasing the oxide loading at constant particle size, although at the expense of losing storage capacity; and (3) for equal oxide loading and particles size, oxides with higher wettability with the salt lead to ss-composites with better anti-leakage efficiency.

The anti-leakage performance of composites Li_4_(OH)_3_Br/MgO, Li_4_(OH)_3_Br/CuO and Li_4_(OH)_3_Br/Fe_2_O_3_ was analyzed, as described in Section 2.2 (step 2 of the screening). For each composite, different pellets (13 mm in diameter, 5 mm in thickness) with oxide content ranging from 20 wt.% to 60 wt.% were prepared in order to determine the maximum salt loading allowed.

The following are shown in Table 4:

- Li_4_(OH)_3_Br/MgO composite shows a minor salt leakage at 30–40 wt.% content of MgO. The sample with 50 wt.% MgO presents no sign of salt leakage, and the pellet shape is perfectly preserved showing a smooth surface without cracks. The sample with 60 wt.% MgO shows good structural stability without salt leakage; however, the pellet has cracked after sintering, which could be due to the high amount of MgO nanoparticles and the lesser amount of the salt, which ensure structural bonding after solidification. The advantages expected of using nanostructure MgO powder with 100 nm particle size were to have shape stabilization at a small loading of MgO thanks to the high specific surface area of MgO nanopowder, which generates a high surface tension between the salt and MgO; however, despite the nanometric particle size used, the form stability was ensured at a minimum content of 50 wt.% MgO, unlike Li_4_(OH)_3_Br/Fe_2_O_3_, and this could be due to the fact that the wettability of MgO by the molten salt is not as high as in the case of Fe_2_O_3_ micropowder.
- Li_4_(OH)_3_Br/Fe_2_O_3_ composite presents a significant salt leakage at 20 wt.% of Fe_2_O_3_. The samples with 30/40/50 wt.% Fe_2_O_3_ present excellent structural stability without any signs of salt leakage. Even though the nanostructure supporting materials prove to afford good anti-leakage efficiency of ss-composite at lesser content compared to materials with micrometric particle size, Fe_2_O_3_ with particle sizes <5 µm shows an excellent structural stability at only 30 wt.% loading compared to 50 wt.% MgO with a particle size of 100 nm. This can indicate the excellent wettability of Fe_2_O_3_ microparticles by the molten salt. In order to afford the maximum enthalpy of phase transition, the minimum content of 30 wt.% Fe_2_O_3_ was chosen for further investigations.
- Li_4_(OH)_3_Br/CuO composite shows a constant improvement of the structural stability and no sign of leakage while increasing the content of the CuO from 30 to 60 wt.%. Samples with 30–40 wt.% of CuO show a significant amount of salt leakage with segregation of salt after sintering, which could be due to the difference in density of the two components. At 50 wt.% CuO, a small leakage of the salt can be observed. While increasing the CuO loading up to 60 wt.%, the shape stabilization is perfectly ensured and no salt leakage was observed. The high CuO loading (60 wt.%) required for the shape stability of the composite could be explained by (i) the large particle size of this material (<74 µm) giving a smaller surface area and thus less surface tension between the molten peritectic salt and CuO required for liquid salt retention inside the structure of the composite; (ii) and/or the modest wettability of CuO by the molten salt. The minimum loading required to guarantee the shape stabilization of the composite is 60 wt.% CuO, although this is at the expense of salt loading. This quite large amount of the supporting material will decrease considerably the storage capacity of the composite. The result was not satisfactory from a thermal storage application point of view, and for this reason, CuO was discarded at this level.

### 3.3. Thermal and Microstructural Characterization and Stability of Li_4_(OH)_3_Br-Based Shape Stabilized Composites

The composites (70Li_4_(OH)_3_Br-30Fe_2_O_3_; 50Li_4_(OH)_3_Br-50MgO), which satisfied the criterion of form stabilization, were characterized by DSC. The objective was to investigate the influence of the shape stabilization on the thermal properties of the salt.

**Figure 5:**
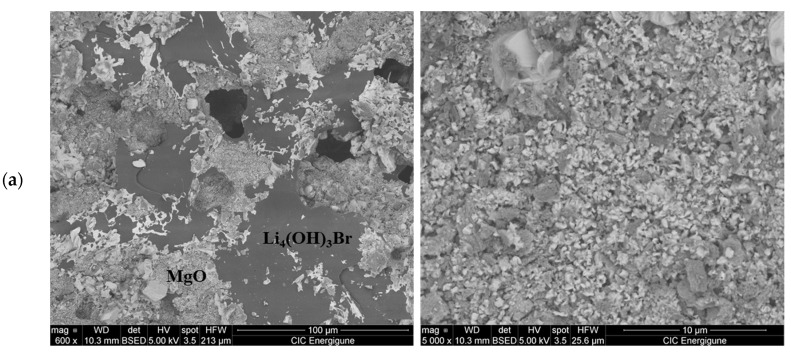

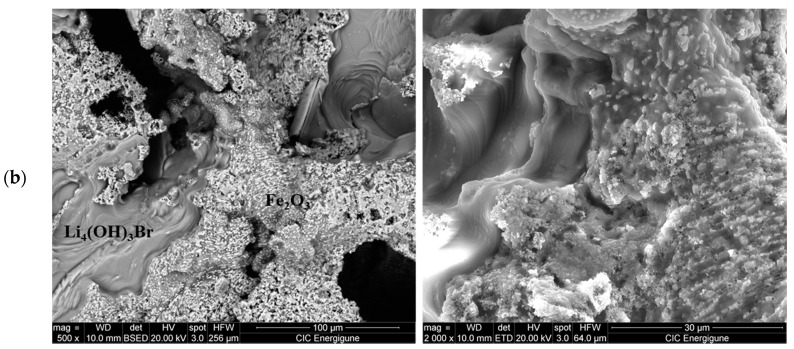
SEM images of the ss-composite: (**a**) 50Li_4_(OH)_3_Br-50MgO; (**b**) 70Li_4_(OH)_3_Br-30Fe_2_O_3._

The morphology of the ss-composites was investigated by SEM. Figure 5a,b present the microstructures of 50Li_4_(OH)_3_Br-50MgO and 70Li_4_(OH)_3_Br-30Fe_2_O_3_ composites respectively. In both cases, it can be seen that Li_4_(OH)_3_Br salt is embedded in the oxide particles. This morphology prevents the leakage of the molten salt outside the structure of the composite by capillary force and surface tension. Analyzing the SEM images, two regions can be distinguished, one region with a smooth surface presenting smooth lamellar undulations which correspond to the peritectic salt, and another region with granular morphology corresponding to the oxide particles. Both structures of the composites present an open porosity.

The results of the DSC in terms of transition temperatures and reaction enthalpies are reported in Table 5.

**Figure 6:**
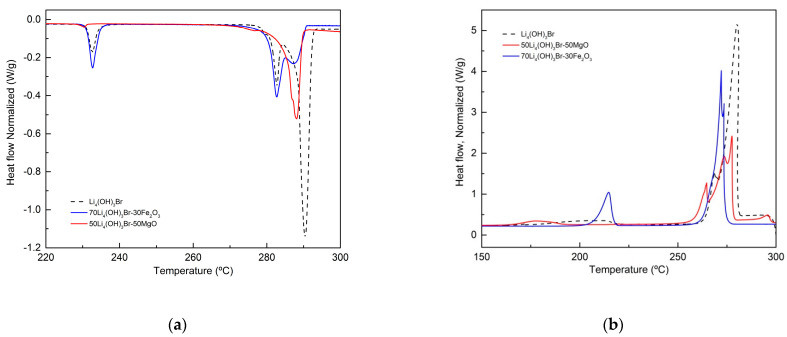
DSC curves of Li_4_(OH)_3_Br-based shape stabilized composites (**a**) upon heating and (**b**) upon cooling.

Both DSC curves of 70Li_4_(OH)_3_Br-30Fe_2_O_3_ presented in Figure 6 and of 90Li_4_(OH)_3_Br-10Fe_2_O_3_ presented in Figure 3 show a shift in the peritectic transition to lower temperatures; the shift increases with increasing Fe_2_O_3_ content (peritectic transition temperature is 288 °C and 281 °C for 90Li_4_(OH)_3_Br-10Fe_2_O_3_ and 70Li_4_(OH)_3_Br-30Fe_2_O_3_, respectively). This can be explained by the possible salt confinement in the interparticle voids of the composites (thanks to the great wettability of Fe_2_O_3_ by the molten salt), which increases with the augmentation of the salt loading. A drop in the enthalpy of the peritectic reaction is also noticed, showing a higher loss for the sample with higher Fe_2_O_3_ loading (10% and 17% enthalpy loss for 90Li_4_(OH)_3_Br-10Fe_2_O_3_ and 70Li_4_(OH)_3_Br-30Fe_2_O_3_, respectively). This can be explained by the fact that the salt trapped inside the interparticle voids of the composite does not contribute to the total reaction enthalpy. Considering the high surface area of MgO compared to Fe_2_O_3_ (particle size is 100 nm and <5 µm for MgO and Fe_2_O_3_, respectively), the aforementioned phenomena is expected to be more pronounced; however, due to the moderate wettability of MgO by the molten salt, the Gibbs–Thomson effect is less likely to occur.

Analyzing Table 5, the most promising material is the composite with MgO as support. A slight enthalpy loss is observed, namely 4% compared to the 17% for the composite with Fe_2_O_3_. This enthalpy loss observed could be a sign of inhomogeneity of the composite structure, which means that the sample analyzed by DSC contained more supporting material than the active material (salt).

**Figure 7:**
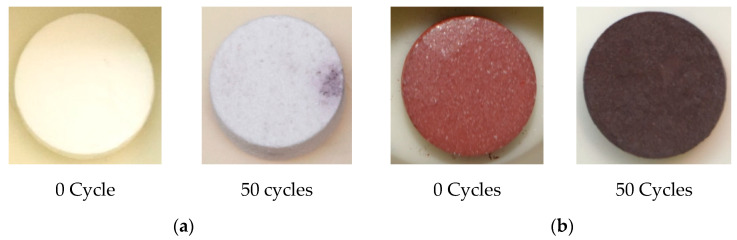
Images of the composite of Li_4_(OH)_3_Br-based shape stabilized composite before and after thermal cycling tests: (**a**) 50Li_4_(OH)_3_Br-50MgO; (**b**) 70Li_4_(OH)_3_Br-30Fe_2_O_3_.

The composites with the best performances (with 50 wt.% of MgO and 30 wt.% of Fe_2_O_3_) were subjected to a lifecycle analysis test (up to 50 cycles) to determine the reactivity and shape stabilization performance after prolonged charge–discharge cycles. The results of these experiments are reported in Figure 7.

No leaks of the salt outside the composite were observed after 50 charge/discharge cycles for both samples. These results are very promising; the number of cycles applied could indeed be considered representative regarding the stability of the shape-stabilized composite. When considering the chemical stability, both composites confirmed the trend already observed after one cycle.

**Figure 8:**
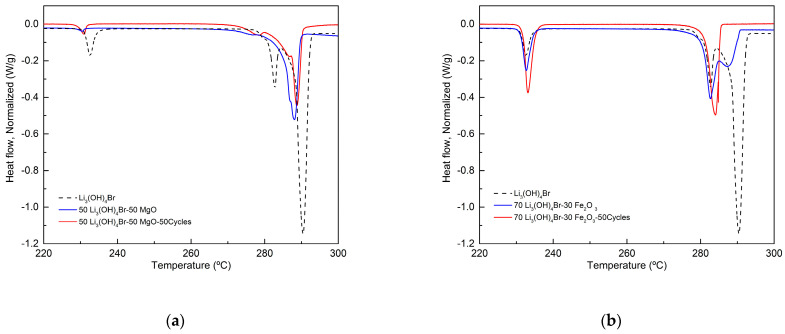
DSC curves of Li_4_(OH)_3_Br-based shape stabilized composites after cycling stability tests: (**a**) 50Li_4_(OH)_3_Br-50MgO; (**b**) 70Li_4_(OH)_3_Br-30Fe_2_O_3_.

The MgO-based composite was stable and inert, as confirmed by the results relevant to the reaction energy after 50 cycles (see Table 5) with enthalpy loss of only 0.5%. The heating DSC curves of 50Li_4_(OH)_3_Br-50MgO composites showed good reproducibility, as can be seen in Figure 8a. On the other hand, the Fe_2_O_3_-based composite showed a significant decrease in the reaction energy of 33%, which excludes its possible further utilization. This result was probably due to a slow and progressive reaction of the salt with the oxide, because of the strong interaction between the two materials, ending with the gradual degradation of the salt. This was manifested by a change in the heating DSC curves, as presented in Figure 8b after 50 heating/cooling cycles.

## 4. Conclusions and Perspectives

The work carried out in this study allowed us to perform the selection of the most promising oxide-based supporting materials, with the best shape-stabilized performances, for the peritectic compound Li_4_(OH)_3_Br for application in thermal energy storage (TES). The combination of thermal treatment, structural analysis, thermodynamic characterization and cycling tests allowed us to have a clear overview of (i) the compatibility of the different materials tested and the possible by-products formed in terms of reacting behavior upon heating; (ii) the influence of the ceramic materials added on the energy of the peritectic reaction; (iii) the shape stabilization capability as a function of the concentration of inert oxide; and (iv) the effect of the cycling tests (up to 50 cycles) on both the reaction energy and the leakage occurrence. The results allowed us to select MgO as the most promising oxide showing good behavior for the two parameters considered. All the other oxides studied showed some type of reaction (more or less pronounced upon heating and upon cycling), leading to their rejection as possible candidate supporting materials. Even though the performance of MgO is good, the high amount used (50 wt.%) in the composite causes a considerable loss of energy density (only half of the reaction energy is available). For this reason, further work is now proceeding to apply different strategies to find new routes to decrease the amount used for maintaining the same performance.

## Figures and Tables

**Table 1:** Main storage-related properties of Li_4_(OH)_3_Br [60].

Peritectic Temperature (°C)	289
Melting point (°C)	340
Thermal conductivity at room temperature (W/m/K)	0.47
Specific heat in solid close to the peritectic temperature (J/g/K)	1.68
Density in solid close to the peritectic temperature (g/cc)	1.85

**Table 2:** General information about the tested oxides.

Material	MgO	Fe_2_O_3_	CuO	SiO_2_	Al_2_O_3_
Supplier	Alfa Aesar Kandel, Germany	Sigma Aldrich St. Louis, MO, USA	Alfa Aesar Kandel, Germany	Sigma Aldrich St. Louis, MO, USA	Sigma Aldrich St. Louis, MO, USA
CAS number	1309-48-4	1309-37-1	1317-38-0	7631-86-9	1344-28-1
Purity (%)	99+%	≥99%	99.7%	>95%	
Particle size	100 nm	<5 µm	<74 µm	12 nm	13 nm
ρ (g/cm^3^)	3.58	5.12	6.315	2.2–2.6	3.95

**Table 3:** Temperatures and enthalpies corresponding to the peritectic reaction of different Li_4_(OH)_3_Br/oxide mixtures after compatibility tests.

Composition	T_onset_ (°C)	Δ*H*_Experimental_ (J/g)	Δ*H*_Calculated_ (J/g)	Enthalpy Loss (%)
Pure Li_4_(OH)_3_Br	289	247	247	
90Li_4_(OH)_3_Br-10Fe_2_O_3_	288	197	222	10
90Li_4_(OH)_3_Br-10CuO	287	215		3
90Li_4_(OH)_3_Br-10MgO	288	209		6
90Li_4_(OH)_3_Br-10Al_2_O_3_	282	137		34

**Table 4:** Li_4_(OH)_3_Br-based ss-composites with different oxide loading after sintering showing salt leakage assessment.

wt.% Oxide	20	30	40	50	60
Li_4_(OH)_3_Br/MgO		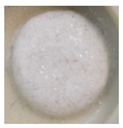	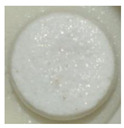	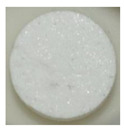	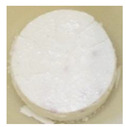
leakage assessment		Serious	Minor	No	No
Li_4_(OH)_3_Br/CuO		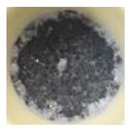	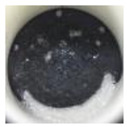	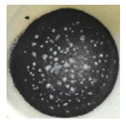	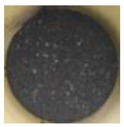
leakage assessment		Serious	Serious	Minor	No
Li_4_(OH)_3_Br/Fe_2_O_3_	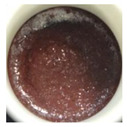	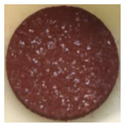	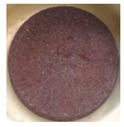	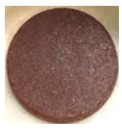	
leakage assessment	Serious	No	No	No	

**Table 5:** Temperatures and enthalpies corresponding to the peritectic reaction of different Li_4_(OH)_3_Br-based ss-composites before and after thermal cycling.

Composition	T_onset_ (°C)	ΔH_Experimental_ (J/g)	ΔH_Calculated_ (J/g)	Enthalpy Loss (%)
Pure Li_4_(OH)_3_Br	289	247	247	
70Li_4_(OH)_3_Br-30Fe_2_O_3_-0Cycle	281	132	173	17
70Li_4_(OH)_3_Br-30Fe_2_O_3_-50Cycles	282	93	173	33
50Li_4_(OH)_3_Br-50MgO-0Cycle	285	114	124	4
50Li_4_(OH)_3_Br-50MgO-50Cycles	287	123	124	0.5
